# Possible Prognostic Value of Serial Brain MRIs in Powassan Virus Encephalitis 

**DOI:** 10.3201/eid2510.181262

**Published:** 2019-10

**Authors:** Joshua Allgaier, Ryan Quarles, Daniel Skiest

**Affiliations:** Baystate Medical Center, Springfield, Massachusetts, USA

**Keywords:** Powassan virus, flavivirus, deer tick encephalitis, United States, viruses, magnetic resonance imaging, MRI, serial brain MRI, meningitis/encephalitis

## Abstract

Powassan virus (POWV) encephalitis is a rare tickborne illness. We describe the clinical course, laboratory findings, and imaging for a patient with POWV in Massachusetts, USA. Clinical presentation and laboratory findings were nonspecific. Improvement on brain magnetic resonance imaging after 2 weeks preceded clinical improvement by months, suggesting possible prognostic value.

Powassan virus (POWV) is a tickborne virus that can cause disease in humans, sometimes in the form of encephalitis. Although rare, encephalitis caused by this virus has been increasingly recognized, especially in the New England and northern Midwest regions of the United States. Clinical course, laboratory findings, and imaging findings are variable, with a few commonly seen trends. Antibody testing diagnoses POWV, but this test is currently done only by the US Centers for Disease Control and Prevention. Treatment options are primarily supportive; no prognostic indicators have been described. We describe the clinical course of POWV encephalitis in a man living in Massachusetts. 

## The Case

A 55-year-old male truck driver with no major medical history developed acute onset of confusion preceded by 2 days of nausea, vomiting, and headache in November 2017. He was found by his co-workers driving his truck in circles and was brought to the hospital by his family, whom he reportedly did not recognize. The patient denied substance abuse or recent travel but was an avid hunter with multiple recent tick bites. Initial vital signs were temperature 101.7°F, blood pressure 124/73 mm Hg, and heart rate 80 bpm. His neck was supple, and he had no rash or focal neurologic deficits.

Hematologic laboratory values were leukocytes 12.5 × 10^9^ cells/mL (reference range 4–11 × 10^9^ cells/mL) with 9.1 × 10^9^ cells/mL neutrophils (reference range 1.3–7.0 × 10^9^ cells/mL); notable metabolic laboratory values were blood urea nitrogen 27 mg/dL (reference range 6–20 mg/dL) and creatinine 1.3 mg/dL (reference range 0.7–1.2 mg/dL). Levels of ammonia, copper, B_12_, and carbon monoxide, as well as liver and thyroid function, were normal. Serologic results were negative for Lyme disease, tularemia, West Nile virus (WNV), HIV, and eastern equine encephalitis virus. Serum PCR results were negative for ehrlichiosis and anaplasmosis. Cerebrospinal fluid (CSF) showed elevated protein (64 mg/dL) and 88 leukocytes/mm^3^ (4% neutrophils, 84% lymphocytes, and 12% monocytes). CSF testing with the FilmArray meningitis/encephalitis panel (BioFire Diagnostics, https://www.biofiredx.com) was negative for DNA of *Escherichia coli* K1, *Haemophilus influenzae*, *Listeria monocytogenes*, *Neisseria meningitides*, *Streptococcus agalactiae*, *Streptococcus pneumoniae*, cytomegalovirus, enterovirus, herpes simplex virus-1 and -2, human herpesvirus-6, human parechovirus, varicella zoster virus, and *Cryptococcus neoformans/gattii*. CSF cytology revealed no abnormalities.

After a largely negative workup for the patient’s unusual memory deficit, we performed brain magnetic resonance imaging (MRI) with and without contrast; results showed symmetric T2 hyperintensities in the bilateral caudate, putamen, and hippocampus, nonspecific findings suggestive of inflammatory encephalitis (Figure). Diffusion-weighted imaging also showed enhancement of the hippocampus (data not shown). These studies were completed during the patient’s ongoing memory deficit and fever with persistent lack of other neurologic findings on examination. A video electroencephalogram identified temporal lobe seizures with mild to moderate generalized background slowing.

**Figure:**
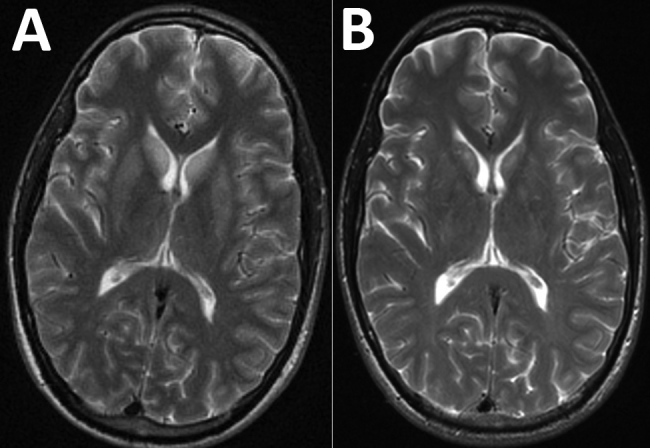
Magnetic resonance imaging (MRI) of the brain of a patient with encephalitis caused by Powassan virus, Massachusetts, USA, 2017. A) Initial brain MRI showing high T2 signal abnormality in the bilateral caudate and putamen. B) Noticeable improvement on repeat brain MRI 2 weeks later.

We treated the patient empirically for bacterial meningitis with ceftriaxone (2 g every 12 h) and for tickborne illness with doxycycline (100 mg every 12 h). When cultures of blood and CSF remained sterile for 48 hours, we stopped all antimicrobial drugs. Over the next 2 weeks, the patient’s memory improved, including better recognition of staff and family. A repeat MRI showed improvement of the previously seen T2 hyperintensities (Figure) and resolution of the hippocampal enhancement on diffusion-weighted imaging (data not shown). The patient was discharged to home the next week with a persistent short-term memory deficit, requiring 24-hour supervision.

One month after discharge, POWV-specific IgM and plaque reduction neutralization tests of the CSF and serum (performed at CDC) confirmed infection. Five months after initial hospitalization, the patient returned to the neurologist, who reported that the patient’s mental status had returned to baseline.

## Conclusions

Since POWV was isolated in Powassan, Ontario, in 1958, just over 100 cases have been described (*1**–**4*). POWV is a flavivirus with 2 serologically indistinguishable lineages (*1**,**2*). Lineage 1 is isolated predominantly from *Ixodes cookei* ticks and lineage 2 (deer tick virus) is isolated predominantly from *I. scapularis* ticks (*2**,**5*). Rarely, POWV has been isolated from other tick species, such as *I. marxi*, *I. spinipalpus*, and *Dermacentor andersoni* (*3*). Other than in humans, evidence of infection has been documented in woodchucks and 37 other mammal species, including red squirrels, chipmunks, and skunks (*2**,**4*). The virus has been detected from Virginia to Nova Scotia, Canada, and in Michigan, Wisconsin, and Minnesota (lineage 1) (*2*). More recently, human cases have been increasingly reported in New England (lineage 2) (*4*).

The clinical course and outcomes of POWV infection are variable and nonspecific. After an incubation period of 1–5 weeks, the most common clinical manifestation is a febrile illness with sore throat, drowsiness, headache, and disorientation (*2*). Other manifestations include rash; gastrointestinal symptoms (*4*); or encephalitis manifesting as vomiting, prolonged fever, respiratory distress, discoordination, difficulty speaking, and seizures (*2*). CSF findings are generally nonspecific and often include elevated protein and lymphocytic pleocytosis (*4*). MRI findings often show T2/FLAIR abnormalities commonly affecting the basal ganglia and thalamus, with noncontiguous lesions in the brainstem, cortex, and periventricular white matter (*2**,**4*). In some cases, brain MRI has been normal, whereas others have reported atypical findings such as microhemorrhages (*4*). Initial MRI findings are sometimes consistent with eventual clinical outcomes, but no definitive correlation has been demonstrated (*4*). Follow-up brain MRI has not been studied previously, and no case reports include mention of evolution of lesions seen on MRI. 

Detection of virus-specific IgM- and IgG-neutralizing antibodies of serum or CSF diagnoses POWV infection (*6*). Viremia usually resolves before encephalitis symptoms, possibly implicating the immune response as a likely cause of clinical manifestations. Approximately 10%–15% of cases with POWV-associated encephalitis are fatal (*1*). Long-term neurologic deficits persist in about half of survivors (*4*). There are isolated case reports of lower mortality with high-dose corticosteroids; however, the number of reported cases is low, and thus no correlation with outcomes has been determined (*2**,**4*). Similarly, the use of intravenous immunoglobulin has been reported, but with minimal apparent impact on outcomes (*2**,**4*).

WNV is a better-understood flavivirus that shares similarities with POWV. Both can manifest as nonspecific encephalitis that can be clinically indistinguishable from each other and with nonspecific CSF findings, usually lymphocytic pleocytosis (*7*). Both WNV and POWV patients show MRI abnormalities predominantly in the thalamus, basal ganglia, and brainstem. Outcomes are similar regarding potential for long-term neurologic deficits and death. Among reported WNV patients, <1% develop meningoencephalitis, but 10% of those develop flaccid paralysis, with a 10% death rate (*7**–**9*). In the few previous case reports of WNV meningoencephalitis that report serial brain MRIs, persistent MRI abnormalities in the posterior fossa were associated with poor outcomes; 1 patient with bilateral edema and hyperintensity of the basal ganglia and thalamus on initial MRI later improved both on MRI and clinically (*9**,**10*). Although a correlation of serial MRI findings with clinical outcomes cannot be concluded from these few previous case reports and our report, they suggest the possibility of prognostic value of serial MRI.

The case we describe is typical of reported cases of POWV encephalitis: nonspecific cognitive impairment, elevated CSF protein and lymphocytic pleocytosis, and T2 hyperintense lesions on brain MRI. The improvement in MRI at 2 weeks preceded our patient’s clinical improvement, suggesting that repeat MRI might have prognostic value. Clinicians in New England and North Central states should consider POWV as a possible etiology in patients with encephalitis in late spring through the fall, during seasonal tick activity.
